# Supplementary material for: Evaluation of nanopore sequencing for increasing accessibility of eDNA studies in biodiverse countries
Source: PLoS One. 2025 Oct 16;20(10):e0333994. doi: 10.1371/journal.pone.0333994 (PMC12530551; doi:10.1371/journal.pone.0333994)
Supplement: Attachment — Submitted filename: PONE-D-25-24113 (1)-reviewed.pdf [file pone.0333994.s005.pdf]

PLOS ONE

Evaluation of nanopore sequencing for increasing accessibility of eDNA studies in biodiverse countries

--Manuscript Draft--

**Manuscript Number:**	PONE-D-25-24113
**Article Type:**	Research Article
**Full Title:**	Evaluation of nanopore sequencing for increasing accessibility of eDNA studies in biodiverse countries
**Short Title:**	Nanopore sequencing for eDNA studies in biodiverse countries
**Corresponding Author:**	Daniel Gygax<br>Technical University Munich: Technische Universitat Munchen<br>GERMANY
**Keywords:**	eDNA, portable sequencing, vertebrates, Zambia, metabarcoding
**Abstract:**	Biodiversity loss is a global challenge of the 21st century. Environmental DNA (eDNA)-based metabarcoding offers a cost- and time-efficient alternative to conventional biodiversity surveys, enabling detection of rare, cryptic, and elusive species from environmental samples. However, limited access to genomic technologies restricts the application of eDNA metabarcoding in highly biodiverse remote regions and low- and middle-income countries. Here, we directly compared the latest portable nanopore sequencing methods with established Illumina sequencing for vertebrate eDNA metabarcoding of Zambian water samples. We also evaluated cost-effective versus established water filtration approaches, and contrasted a comprehensive, computationally intensive taxonomic database search with a streamlined, manually curated database search. Our results show that due to recent improvements in sequencing chemistry and optimized basecalling, nanopore sequencing can recover many of the species detected by Illumina sequencing, demonstrating the feasibility of in situ biodiversity assessment using portable platforms. We, however, found that highly accurate Illumina sequencing remains superior in the quantity of taxonomic detections, and that eDNA- and camera trap-based species detections had minimal overlap, suggesting a complementary rather than substituting application of these biodiversity monitoring technologies. Finally, our findings underscore the role of database completeness for taxonomic assignments, emphasizing the need for high-quality ecosystem-specific reference databases in eDNA research.
**Order of Authors:**	Daniel Gygax
	Sabina Ramirez
	Moses Chibesa
	Twakundine Simpamba
	Michael Riffel
	Tom Riffel
	Amrita Srivathsan
	Reindert Nijland
	Lara Urban
**Additional Information:**	
**Question**	**Response**
**Financial Disclosure**<br><br>Enter a financial disclosure statement that describes the sources of funding for the work included in this submission. Review	The author(s) received no specific funding for this work.

Powered by Editorial Manager® and ProduXion Manager® from Aries Systems Corporation

the submission guidelines for detailed requirements. View published research articles from *PLOS ONE* for specific examples.<br><br>This statement is required for submission and **will appear in the published article** if the submission is accepted. Please make sure it is accurate.<br><br>**Funded studies**<br>Enter a statement with the following details:<br>• Initials of the authors who received each award<br>• Grant numbers awarded to each author<br>• The full name of each funder<br>• URL of each funder website<br>• Did the sponsors or funders play any role in the study design, data collection and analysis, decision to publish, or preparation of the manuscript?<br><br>Did you receive funding for this work?	
**Competing Interests**<br><br>Use the instructions below to enter a competing interest statement for this submission. On behalf of all authors, disclose any competing interests that could be perceived to bias this work—acknowledging all financial support and any other relevant financial or non-financial competing interests.<br><br>This statement is required for submission and **will appear in the published article** if the submission is accepted. Please make sure it is accurate and that any funding sources listed in your Funding Information later in the submission form are also declared in your Financial Disclosure statement.<br><br>View published research articles from *PLOS ONE* for specific examples.	Enter: The authors have declared that no competing interests exist.

**NO authors have competing interests**<br><br>Enter: *The authors have declared that no competing interests exist.*<br><br>**Authors with competing interests**<br><br>Enter competing interest details beginning with this statement:<br><br>*I have read the journal's policy and the authors of this manuscript have the following competing interests: [insert competing interests here]*<br><br>* typeset	
**Ethics Statement**<br><br>Enter an ethics statement for this submission. This statement is required if the study involved:<br><br>• Human participants<br>• Human specimens or tissue<br>• Vertebrate animals or cephalopods<br>• Vertebrate embryos or tissues<br>• Field research<br><br>Write "N/A" if the submission does not require an ethics statement.<br><br>General guidance is provided below. Consult the submission guidelines for detailed instructions. **Make sure that all information entered here is included in the Methods section of the manuscript.**	Department of National Parks and Wildlife, Zambia<br>Research permit: NPW/8/27/1

**Format for specific study types**<br><br>**Human Subject Research (involving human participants and/or tissue)**<br>• Give the name of the institutional review board or ethics committee that approved the study<br>• Include the approval number and/or a statement indicating approval of this research<br>• Indicate the form of consent obtained (written/oral) or the reason that consent was not obtained (e.g. the data were analyzed anonymously)<br><br>**Animal Research (involving vertebrate animals, embryos or tissues)**<br>• Provide the name of the Institutional Animal Care and Use Committee (IACUC) or other relevant ethics board that reviewed the study protocol, and indicate whether they approved this research or granted a formal waiver of ethical approval<br>• Include an approval number if one was obtained<br>• If the study involved *non-human primates*, add *additional details* about animal welfare and steps taken to ameliorate suffering<br>• If anesthesia, euthanasia, or any kind of animal sacrifice is part of the study, include briefly which substances and/or methods were applied<br><br>**Field Research**<br><br>Include the following details if this study involves the collection of plant, animal, or other materials from a natural setting:<br>• Field permit number<br>• Name of the institution or relevant body that granted permission	
**Data Availability**<br><br>Authors are required to make all data underlying the findings described fully available, without restriction, and from the time of publication. PLOS allows rare exceptions to address legal and ethical concerns. See the PLOS Data Policy and FAQ for detailed information.	Yes - all data are fully available without restriction

A Data Availability Statement describing where the data can be found is required at submission. Your answers to this question constitute the Data Availability Statement and **will be published in the article**, if accepted.<br><br>**Important:** Stating 'data available on request from the author' is not sufficient. If your data are only available upon request, select 'No' for the first question and explain your exceptional situation in the text box.<br><br>Do the authors confirm that all data underlying the findings described in their manuscript are fully available without restriction?	
**Describe where the data may be found in full sentences. If you are copying our sample text, replace any instances of XXX with the appropriate details.**<br><br>• If the data are **held or will be held in a public repository**, include URLs, accession numbers or DOIs. If this information will only be available after acceptance, indicate this by ticking the box below. For example: *All XXX files are available from the XXX database (accession number(s) XXX, XXX.*).<br>• If the data are all contained **within the manuscript and/or Supporting Information files**, enter the following: *All relevant data are within the manuscript and its Supporting Information files.*<br>• If neither of these applies but you are able to provide **details of access elsewhere**, with or without limitations, please do so. For example:<br><br>*Data cannot be shared publicly because of [XXX]. Data are available from the XXX Institutional Data Access / Ethics Committee (contact via XXX) for researchers who meet the criteria for access to confidential data.*<br><br>*The data underlying the results presented in the study are available from (include the name of the third party*	All sequencing data is deposited in the NCBI Sequence Read Archive (SRA; accession number: PRJNA1195924).<br>The code used for this study is available 457 at: https://github.com/dmgr90/eDNA-Zambia-1

and contact information or URL).<br>• This text is appropriate if the data are owned by a third party and authors do not have permission to share the data.<br><br>* typeset	
Additional data availability information:	

Powered by Editorial Manager® and ProduXion Manager® from Aries Systems Corporation

Manuscript

Click here to access/download;Manuscript;Manuscript Zambia eDNA Final .pdf

Evaluation of nanopore sequencing for increasing accessibility of eDNA studies in biodiverse countries

Daniel Gygax[1,5]*, Sabina Ramirez[1], Moses Chibesa[2], Twakundine Simpamba[7], Michael Riffel[3], Tom Riffel[6], Amrita Srivathsan[8], Reindert Nijland[9], Lara Urban[4,5]*

*Corresponding authors: dgygax90@gmail.com & lara.h.urban@gmail.com

1Faunomics GmbH

2Department of Zoology and Aquatic Sciences, The Copperbelt University

3Rifcon GmbH

4Technical University of Munich, School of Life Sciences

5Helmholtz Munich

6Nsanga Conservation Zambia

7Department of National Parks and Wildlife Zambia

8Center for Integrative Biodiversity Discovery, Museum für Naturkunde Berlin

9Marine Animal Ecology Group, Wageningen University

Abstract

Biodiversity loss is a global challenge of the 21st century. Environmental DNA (eDNA)-based metabarcoding offers a cost- and time-efficient alternative to conventional biodiversity surveys, enabling detection of rare, cryptic, and elusive species from environmental samples. However, limited access to genomic technologies restricts the application of eDNA metabarcoding in highly biodiverse remote regions and low- and middle-income countries. Here, we directly compared the latest portable nanopore sequencing methods with established Illumina sequencing for vertebrate eDNA metabarcoding of Zambian water samples. We also evaluated cost-effective versus established water filtration approaches, and contrasted a comprehensive, computationally intensive taxonomic database search with a streamlined,

manually curated database search. Our results show that due to recent improvements in sequencing chemistry and optimized basecalling, nanopore sequencing can recover many of the species detected by Illumina sequencing, demonstrating the feasibility of in situ biodiversity assessment using portable platforms. We, however, found that highly accurate Illumina sequencing remains superior in the quantity of taxonomic detections, and that eDNA- and camera trap-based species detections had minimal overlap, suggesting a complementary rather than substituting application of these biodiversity monitoring technologies. Finally, our findings underscore the role of database completeness for taxonomic assignments, emphasizing the need for high-quality ecosystem-specific reference databases in eDNA research.

Keywords

eDNA, portable sequencing, vertebrates, Zambia, metabarcoding

Introduction

Biodiversity loss is one of the major challenges of the 21st century [1], but efforts to prevent biodiversity loss are often hindered by a lack of knowledge of species distributions and dynamics [2][3]. Environmental DNA (eDNA) approaches can provide a cost- and time-efficient alternative to conventional biodiversity monitoring methods by detecting taxa, including rare, cryptic, and elusive species, from metabarcoding their genetic material in environmental samples such as water, soil, or air, and subsequent amplicon sequencing [4][5][6]. Such eDNA approaches have recently become cheaper and more reliable in monitoring the temporal and spatial distribution and dynamics of species, populations, and communities [7][8][9]. The lack of access to genomic technology, however, hinders the application of metabarcoding approaches in remote areas and low- and middle-income countries (LMICs), which often contain the globally largest amount of biodiversity [10][11][12]. Simultaneously, international treaties such as the Convention on International Trade in Endangered Species of Wild Fauna and Flora [13] and data sovereignty principles as specified by the Nagoya Protocol on Access and Benefit Sharing [14] and the Global Indigenous Data Alliance [15] often require the generation and usage of such genomic data close to the species' origin.

Long-read nanopore sequencing technology as developed by Oxford Nanopore Technologies (ONT) is the first genomic technology that enables rapid *in situ* sequencing at low upfront investment costs through its portable devices. This technology therefore holds the promise of putting eDNA research in line with international treaties and data sovereignty principles, while saving time and resources otherwise spent on sample transport and exportation, shortening the gap between research and decision-making and potentially empowering local researchers, conservationists, and decision-makers [11][16]. First applications of nanopore sequencing to metabarcoding in general [17][18][19] and eDNA specifically [20] have shown promising results in species but in comparison to the established sequencing platform for metabarcoding, short-read Illumina sequencing, remained hampered by the relatively high sequencing error rates. [18] have, however, also shown that this lack in sequencing accuracy can be

compensated by leveraging nanopore sequencing's long-read capability, which enabled species-level resolution in their protistan studies by substantially increasing the targeted amplicon size. While the error rate of this previously applied nanopore sequencing chemistry (“R9” chemistry) was estimated at anywhere between 5% and 22% [17][19], recent substantial improvements in the nanopore design (“R10” chemistry) and basecalling algorithms now allow for highly accurate nanopore read- and consensus-level accuracy. [2] were the first to leverage these accuracy improvements to apply nanopore-based eDNA to successfully monitor marine vertebrates from aquarium and sea water; similar to [18] they showed that an increase of the targeted amplicon size also improves species detection when using the latest nanopore chemistry, but that fragmentation of the marine vertebrates’ DNA in real-world environments such as the sea hampers the amplification of long stretches of DNA [21].

Here, we conducted an eDNA study to directly compare the latest improvements in portable nanopore sequencing with established Illumina sequencing for monitoring vertebrate diversity from freshwater samples. To facilitate *in situ* eDNA research, we further compared established with more cost-effective water filtering methods, and a computationally intensive full taxonomic database search with a locally executable manually annotated database search. We specifically applied our protocols for vertebrate monitoring in Zambia with a focus on terrestrial biodiversity: This country is a good example of a biodiversity rich country that shares borders and ecosystems with eight other African countries and is estimated to be home to 242 mammals, 757 bird, 74 amphibian, 156 reptile, and 490 fish species [22] Zambia has further been described more as “a large patchwork of important ecosystems” than a biodiversity hotspot. The subtropical savannahs ecosystems are characterized by extreme oscillations of precipitation and water availability during the wet and dry seasons [23][24]; this results in an increased reliance on mobility for both humans and animals to access water [24] which imposes challenges for monitoring highly mobile animals across large areas. The fast *in situ* application of eDNA to vertebrate diversity monitoring by sampling freshwater and applying

portable nanopore sequencing could therefore provide a practical alternative for tracking highly vagile and illusive vertebrates in the Zambian subtropical savannahs.

Materials and Methods

Environmental DNA collection

Freshwater sampling was conducted at five locations in Luambe and Lukusuzi National Parks within the Luangwa Valley in Eastern Zambia between July 13th and 16th 2023. In Luambe National Park, three water bodies were sampled: an artificial pond near an established tourism facility (CAM: 12.45880° S, 32.14662° E) and two permanent natural ponds (P2: 12.48503° S, 32.17606° E; P3: 12.48246° S, 32.19048° E). In Lukusuzi National Park, samples were collected from two sites along the Lukusuzi River (L1: 12.71192° S, 32.50254° E; L2: 12.71247° S, 32.50226° E). These locations were chosen because previous camera trap-based monitoring had identified them as hotspots of vertebrate activity.

Freshwater samples were collected using two filtering methods. In the first method, 1 liter of water per sample was filtered through a standard paper coffee filter (Melitta® 1x4), previously sterilized by placing them under UV light in the extraction hood for one hour. The water was filtered by simply holding down the filter with sterile gloves and pouring the water through. In the second method, 1 liter of water per sample was filtered using a 0.45 µm nitrocellulose filter cup (Thermo Scientific Nalgene, 250 mL) which are routinely used for water eDNA studies, and using a vacuum pump to speed up the process. After filtration, coffee filters were placed in 50 mL Falcon™ tubes and stored in sterile Ziplock bags with silica gel beads. The nitrocellulose filters were first stored in sterile kraft bags, and then placed in sterile ziplock bags with silica gel beads.

At each water body, three biological replicates (A, B, and C) were collected. For samples with high sediment content, multiple nitrocellulose filters were required to filter the entire liter due

to clogging; these filters were processed separately during DNA extraction with DNA extracts subsequently being pooled per replicate.

Environmental DNA extraction

All molecular work was conducted at the Helmholtz Center Munich laboratories in Germany under the corresponding research permits from the Zambian Department of National Parks and Wildlife, (Reference number: NPW/8/27/1). The nitrocellulose filters were cut in half using a sterile scalpel, sterile gloves, and a PF2 mask, then stored separately in 1.5 ml Eppendorf tubes at -20°C until further processing; only half of a filter was then used for DNA extraction while the other half was kept as a backup. For the coffee filter, two 2x3 cm^2 pieces were cut from the lowest portion using the same sterile procedures and storage conditions. DNA was extracted from all samples using the DNeasy PowerSoil Pro Kit (Qiagen) following the manufacturer's instructions, with the exception DNA was eluted in 50 µl of elution buffer in the final step.

For the filter negative controls, 1 liter of sterile Milli-Q® water was filtered through both filter types. An extraction and PCR control were also included where sterile water was used instead of a piece of filter and DNA template, respectively. Together with all biological replicates across all sampling sites, this resulted in 33 DNA extracts, whose DNA concentrations were measured with an Invitrogen™ Qubit™ 3.0 Fluorometer using the High Sensitivity (HS) Assay.

PCR amplification and metabarcoding

Metabarcoding was carried out using the 12S rRNA variable region 5 (vertebrate-specific primer set 12SV05: forward 5′-TTAGATACCCC ACTATGC-3′ and reverse 5′-TAGAACAGGCT CCTCTAG-3′) to amplify a ~97 bp marker region [25]. Each sample extract was amplified in PCR triplicates, and each filter negative control in two PCR replicates each, resulting in overall 96 PCR products. The PCR reactions were set up in 20 µL volumes consisting of 2 µL DNA template, 12.5 µL Phusion® High-Fidelity PCR Master Mix, 1.25 µL of

each primer, and 8 μL of DNase-free water. The cycling parameters were as follows: 95 °C for 10 minutes, followed by 40 cycles of 94 °C for 30 seconds, 60 °C for 30 seconds, and 70 °C for 60 seconds, with a final extension at 72 °C for 7 minutes.

A symmetrical tagged primer approach was used to multiplex the samples during amplification. Unique 9 bp tags, generated using Barcode Generator (https://github.com/lcomai/barcode_generator), were added to the 5′ ends of both the forward and reverse primers for each primer set. This tag length has been shown to be suitable for multiplexing amplicons for nanopore sequencing [26]. All tags were designed to differ by at least 3 bp from each other, and the same tag was used on both the forward and reverse primers of each PCR product.

Following amplification, one replicate PCR product was respectively visualized on 2% agarose gels with GelRed and Invitrogen™ 100 bp DNA. Amplification was confirmed by the presence of a band of the expected size, which was the case for all visualized samples (including the negative controls).

Sequencing library preparation and sequencing

The 96 PCR products were pooled by taking 10 μl of each product, and then cleaned using a 2:1 bead-to-DNA ratio with AMPure XP Beads (Beckman Coulter) followed by resuspension in 100 μL of DNase-free water. This final pool was separated into 50 μL volumes as input for nanopore and Illumina sequencing, respectively.

Nanopore sequencing was performed using the portable MinION Mk1b device. Library preparation was done using the Ligation Sequencing (SQK-LSK114) following the manufacturer’s Ligation Sequencing Kit Amplicons protocol with the following modification: At the end of the protocol, 30 μL of elution buffer was used instead of the specified 15 μL. This was done to prepare two libraries in one preparation, which was possible due to high DNA

input into the library preparation. Both libraries were consecutively sequenced for 16 hours on one flow cell, separated by a flow cell wash step.

Illumina sequencing was performed by Novogene, GmbH, Germany, using their 2x150 bp protocol on a NovaSeq 6000.

Data processing and OTU clustering

Nanopore raw data was basecalled using Dorado’s (v0.7.0) super high accuracy model (SUP; https://github.com/nanoporetech/dorado). The resulting FASTQ files were demultiplexed using OBITools4's obimultiplex command, allowing for a maximum of one error in the tags (https://git.metabarcoding.org/obitools/obitools4). Primers were trimmed using Cutadapt (v4.9) [27]. Subsequent Operational Taxonomic Unit (OTU) processing steps—including filtering, dereplication (with singleton removal), chimera removal, and clustering at a 97% sequence identity threshold—were performed using VSEARCH (v2.28.1) [28].

Illumina data was demultiplexed using Cutadapt (v4.9), allowing for a maximum of one error in the tags. Primers were trimmed using Cutadapt (v4.9), and paired-end reads were then merged using the BBMerge command from BBMap (v39.11) [29]. Subsequent OTU processing steps were identical to the OTU processing steps of the nanopore sequencing data.

To evaluate the impact of read quality filtering on the number of retained reads, the number of OTUs, and the number of species in both sequencing datasets, we repeated the respective processing steps while adjusting the VSEARCH fastq_maxee parameter to thresholds of 0, 0.1, 0.5, and 1.0, respectively: For a 97 bp sequence, fastq_maxee values of 1.0, 0.5, and 0.1 correspond to average per-base quality scores of approximately 20, 23, and 30, respectively. This was calculated using the formula: $Q = -10 \times \log_{10}(\text{fastq\_maxee} \div L)$, where Q is the average per-base Phred quality score and L is the read length. Therefore, lower fastq_maxee

thresholds require higher average per-base quality scores to keep the expected error per read below the specified limit.

Taxonomic assignments

Amphibian, bird, mammalian, and bird species lists reported in Zambia were obtained from the SASCAL portal (http://data.sasscal.org/metadata/view.php?view=doc_documents&id=3154, 2017), while the list of fish species occurring in Zambia was obtained from FishBase (https://fishbase.mnhn.fr/country/CountryCheckList.php?c_code=894). These species lists were used to download all available 12S rRNA and full mitochondrial sequences on NCBI for building our reduced Zambian species only database. To avoid bias and overrepresentation, only a maximum of 5 reference sequences per species were kept in the database. The same species lists were used to assess our study’s database incompleteness database. The full vertebrate species list can be found in Supplementary Table 1.

Taxonomic assignment of the OTUs was then done using BLAST (v2.15.0) [30] against the full NCBI nt database and against the reduced databases containing all available 12S rRNA and full mitochondrial sequences of the “plausible” species, i.e. either vertebrate species reported in Zambia or common domestic species and humans, which could occur as common contaminants (*Homo sapiens*, *Sus scrofa*, *Bos taurus*, *Gallus gallus*, *Ovis aries*, *Felis catus* and *Canis lupus*). The three best taxonomic hits with at least 95% sequence similarity score and 70% query coverage score were taken and then filtered for plausible taxon detections. If multiple Zambian species were found among the three hits, only the one with the highest sequence similarity score was kept. Although a 95% identity threshold is generally considered low, we deemed it appropriate for this study’s objective of comparing sequencing technologies—particularly considering nanopore’s still relatively higher error rate—and because we restricted our analysis to species known to occur in Zambia. However, one limitation of this approach is that not all Zambian species are represented in current

databases, making misidentifications more likely if a query sequence is assigned to the nearest taxon at this lower threshold.

Principal Coordinate Analysis

To assess patterns of compositional dissimilarity among freshwater samples, we conducted a Principal Coordinate Analysis (PCoA) using the Jaccard distance metric based on species presence-absence data to capture qualitative differences in community composition. We first pooled PCR replicate data within each sampling site, sequencing technology, and filter type to create the distance matrix and visualized the major axes of variation using the R vegan package (v2.6-6.1).

Validation through Camera Trapping

One week prior to eDNA sampling, camera traps (SECACAM Wild-Vision Full HD 5.0) were put up at sampling locations to validate species detections in the subsequent freshwater eDNA samples. Cameras, equipped with passive infrared sensors (PIR), were set to picture-mode and recorded for 24 hours. The number of deployed camera traps varied from 2 to 5 cameras across sites, depending on the size and accessibility of the water bodies. Cameras were positioned to cover the entire waterbodies with priority on established drinking places by large mammals. Drinking places were determined by visual assessments on mammal tracks, scat or further signs of presence.

Results

Our database revision revealed that genetic references for vertebrate groups in the NCBI repository are generally limited across all taxa for both the 12S gene and full mitochondrial sequences. Amphibians have 58.14% coverage for the 12S gene, while only 6.98% have full mitochondrial references. Reptiles show 42.78% representation for 12S references and only

3.09% for full mitochondrial sequences. Fish have 23.62% coverage for 12S and 9.30% for full mitochondrial sequences. Mammals are relatively well represented with 58.80% for the 12S gene and 57.60% for full mitochondrial sequences. Birds exhibit 28.38% coverage for 12S and only 15.64% for full mitochondrial sequences. These numbers highlight the considerable gaps in genetic reference data for Zambian amphibians, reptiles, fish (Supplementary Table 2).

Across all freshwater samples, a total of 26,733,749 out of 40,008,573 nanopore sequencing reads (66.8%) were successfully demultiplexed. For Illumina sequencing, 30,461,385 out of 39,136,312 reads (77.8%) were successfully demultiplexed. Samples yielding fewer than 1,000 sequencing reads were excluded. In the Illumina dataset, 14 such samples were excluded, and in the nanopore dataset, two additional samples were excluded. We confirmed that these low-read samples did not produce any operational taxonomic units (OTUs), except for two Illumina samples that each yielded a single out, respectively.

The remaining samples showed uneven read coverage, with between 3,603 and 2,396,646 nanopore reads per sample (mean = 280,582.0; SD = 475,232.25), and between 10,715 and 966,066 Illumina reads per sample (mean = 353,724; SD = 206,444.44). For the negative controls, nanopore read counts ranged from 8,909 to 216,181 (mean = 103,879.0; SD = 90,494.15), while Illumina read counts ranged from 239,821 to 681,569 (mean = 478,349.0; SD = 181,446.6).

Quality filtering based on the expected sequencing error substantially impacted the number of nanopore reads (retention of only ~53% of reads after Q30 filtering), but not of Illumina reads (retention of ~97%; Figure 1A). The same pattern was subsequently detected in the number of OTUs, where nanopore-based OTU counts dropped from 28,305 (Q0) to 14,852 (52.5%) at Q20, 10,035 (35.5%) at Q23, and only 5,314 (18.8%) at Q30, while Illumina-based OTU count were unaffected by the quality threshold cut-offs (Figure 1A). At the species detection level, this pattern was still present, but less pronounced, where the nanopore sequencing dataset

led to the detection of 35 (Q0), 32 (Q20), 31 (Q23), and 27 (Q30) species, while the species count of 40 in the Illumina sequencing data was independent of the quality filtering (Figure 1A).

We proceeded with all analyses at a quality threshold of Q20 for both sequencing datasets since this filter substantially decreased the possibly inflated number of nanopore OTUs while retaining most species detections by both sequencing modalities (Figure 1A).

All five vertebrate classes were detected by nanopore and Illumina sequencing; for both sequencing technologies the highest number of species detections occurred for fish (15 species, respectively) and mammals (10 and 16 species, respectively; Figure 1B; Supplementary Table 3). Across all vertebrate classes, 29 species were detected by both sequencing technologies while eight species could only be detected by the Illumina dataset; in both datasets, there were three cases where a species was detected in one sequencing dataset with a congeneric hit in the respective other dataset (Figure 1C; Supplementary Table 3).

Figure 1. Freshwater sample sequencing results. A) *From top to bottom*: Sequencing read, Operational Taxonomic Unit (OTU), and species counts for nanopore ("ONT", blue) and Illumina (green) and sequencing datasets across multiple quality filtering thresholds (Q0: unfiltered, Q20, Q23, and Q30; Materials and Methods). B) Number of species detected per vertebrate class and order by both sequencing datasets at a Q20 filtering threshold (*left*: ONT; *right*: Illumina). C) Number of species detected by either or both sequencing datasets. The "ONT congeneric" (purple) and "Illumina congeneric" (red) species counts represent the number of species that were detected in one sequencing dataset with a congeneric hit in the respective other dataset.

We found that most species were only detected by a small number of replicates (Table 1). After removing matches to domestic species, several species occurring in Zambia still showed up in the negative controls and were also very often present in many PCR samples. Among these species are species found in the study area such as *Hippoppotamus amphibius* and *Helogale parvula*, the former was also detected by camera traps in this study. Two frog species, *Xenopus laevis* and *Xenopus muelleri*, were detected at varying frequencies. *X. laevis*, was detected in three samples in the nanopore dataset. In contrast, *X. muelleri* appeared in 23 nanopore samples and 82 Illumina samples, as well as in all negative controls. Fish accounted for the taxon with species showing up in the highest number of samples, seven of which appeared also in the negative controls.

Table 1. Summary of species detected by environmental DNA across samples, negative controls and sequencing platforms.

We next assessed the number of species detections across all freshwater samples (Supplementary Table 4). The number of species was similar between the two filter types, the standard nitrocellulose filter and the more cost-efficient coffee filter (Figure 2A), and between the five sampling sites (Figure 2B; Materials and Methods; Supplementary Table 5). The majority of variance in species detections between freshwater samples seems to stem from the sequencing technology, with Illumina sequencing systematically leading to higher species detections (Figure 2A-B ; Supplementary Table 5). Principal coordinate analysis (PCoA) of the Jaccard distance between all freshwater samples with respect to species detections showed that also the majority of variance in species composition across the samples stemmed from the two sequencing technologies: the first PCoA axis, which explains 41.64% of the variance of species composition across samples, clearly separates Illumina- and nanopore-sequenced samples (Figure 2C). Samples belonging to the same filtering methods and from the same

sites, on the other hand, tend to cluster together within each sequencing technology, with some variation explained by the second PCoA axis (Figure 2C).

Figure 2. Species detections across freshwater samples by Illumina and nanopore ("ONT") sequencing. A) Number of species detected by the two filter types ("NC": nitrocellulose filter; "CO": coffee filter) by both sequencing technologies across all PCR replicates. B) Number of species detected across the five sampling sites (CAM, L1, L2, P2, and P3; Materials and Methods) by both sequencing technologies across all PCR replicates. C) Principal coordinate analysis (PCoA) of the Jaccard distance of all freshwater samples after PCR replicate pooling across sampling sites, sequencing technology, and filter types.

We next compared all our species detections with eDNA-based species detections using a reduced database that only contains the plausible species (Materials and Methods), since such an approach holds the promise of being executable rapidly on portable computational machines. As such a reduced-database analysis might, however, lead to an increased number of false-positive species detections, we additionally analyzed camera trap images that were taken at the same sampling sites shortly before freshwater sampling to obtain a true-positive vertebrate species list, focusing on non-fish vertebrate taxa (Materials and Methods; (Supplementary Table 6). Briefly, camera trapping detection was biased towards birds and mammals with 10 and 9 species detected, respectively, and no possible species-level classification in the other vertebrate classes (Supplementary Table 5). We then compared the species detections between our original "full database" with the "reduced database" and the visual camera trap-based species monitoring approach (Figure 3). We found that only four species (Figure 3A), five genera (Figure 3B), and seven families (Figure 3C) were detected by all three methods, and that the reduced-database analysis led to the detection of many

additional species whose presence could mostly not be confirmed by camera trap-based monitoring (Figure 3).

Figure 3. Shared taxa detections between camera trap-based biodiversity monitoring and freshwater-based eDNA-based monitoring using a “full” versus a “reduced” taxonomic database that only contains plausible species: A) Species, B) genus, and C) family detections. D) Examples of camera trap pictures of species detected by all three approaches: *left*: Small-spotted Genet (*Genetta genetta*); *middle*: Emerald-spotted wood dove (*Turtur chalcospilos*); and *right*: Hippopotamus (*Hippopotamus amphibius*).

Discussion

The *in situ* application of eDNA in biodiverse areas and countries is of utmost importance to fulfill international treaties on data sovereignty, material export, and benefit sharing when it comes to genetics-based biodiversity monitoring; established Illumina sequencing for metabarcoding is, however, based on expensive machinery that is mostly only accessible in centralized sequencing facilities or research laboratories. Here, we use freshwater samples from Zambian ecosystems to show that the improvements in portable nanopore sequencing technology through chemistry updates (R10.4.1) and improved basecalling (Dorado SUP) can capture the majority of species discovered by established Illumina sequencing. This shows the potential of applying this portable genomic technology and cost-effective sample multiplexing for *in situ* biodiversity monitoring in the area or country of interest.

We, however, also found that Illumina sequencing of eDNA captured more species than nanopore sequencing (especially when filtering the nanopore data at a high-quality score of Q30), and that the largest variability in species composition and distribution across our freshwater samples was explained by the sequencing technology—and not by the location or filter type of the sample. Illumina sequencing captured six taxonomic families that could not be detected by nanopore sequencing—although three of those six families could be recovered if the sequence mapping identity of the nanopore OTUs was reduced from 95 to 94%. This

suggests that the accuracy of nanopore sequencing still has to be improved to be comparable to highly accurate Illumina sequencing for species profiling using short gene regions.

Due to its long sequencing reads, nanopore sequencing, however, also holds promise for basing metabarcoding studies on longer marker gene regions [18][21][31]. For example, [18] showed that while nanopore reads had a higher error rate (using the R9 nanopore chemistry), the full length 18S rRNA region sequenced with nanopore achieved a greater taxonomic classification accuracy down to the species level in soil protistan communities than the shorter Illumina-sequenced Variable Region 9 of the same gene, which only provided reliable classification on the phylum level. Similarly, [21] (using the R10 nanopore chemistry) compared a universal primer pair targeting a 2 kb region of fish mitochondrial DNA against the commonly used MiFish primer pair targeting a ~170 bp region, concluding that while the longer amplicon allowed for more species assignment in a controlled aquarium condition, the shorter amplicon performed better in natural environmental settings. This is because eDNA is often highly fragmented due to various environmental factors such as UV radiation, microbial activity, and enzymatic degradation, resulting in higher persistence time for shorter fragments [32][33][34], and such shorter DNA fragments are more likely to amplify during PCR reactions [35]. When using eDNA to monitor vertebrates, especially terrestrial vertebrates from freshwater samples like presented in our study, the samples' DNA might therefore be too fragmented to allow for efficient long-read gene sequencing—which remains to be tested, using for example vertebrate-specific full-length amplicon primers. The suitability of amplicon length in metabarcoding studies might ultimately depend on different factors, such as the targeted taxa, the environmental DNA source, and the amount of genetic variability contained within the amplified marker.

Even for small marker gene regions (such as the 12S rRNA variable region 5 as used in this study), genomic databases remain incomplete, especially when it comes to the biodiversity of megadiverse but understudied ecosystems [36][37][38] —such as Zambia's subtropical savannahs ecosystems as assessed in this study. The mapping of marker gene OTUs to

publicly available databases such as the entire NCBI nt database strongly depends on the regional taxonomic coverage, and can lead to unassigned sequences or false assignments in the case of incomplete databases [39][40]. While many eDNA studies rely on post-OTU-mapping filtering of taxonomic hits to plausible species that are known to occur in the geographic area of interest [41][42][43][44], the pre-mapping reduction of databases to customized localized databases potentially results in higher resolution of taxonomically assigned species [40], especially after *in silico* amplification-based reduction and taxonomically aware dereplication [45]. We therefore compared the taxonomic assignments of both Illumina- and nanopore-based OTUs when using a full species database followed by plausible taxon filtering, or when using a reduced database that only contains plausible taxa. We importantly found an incomplete overlap in non-fish vertebrate species detections by both database approaches; this low overlap was mainly due to many OTUs mapping to taxa in the reduced database mapped to non-plausible taxa using the full-database approach. In the few instances (n=7) were an OTU mapped to a plausible taxon only in the full database, this taxon was always a species closely related to any of the plausible species; the plausible taxa only detected by the full-database approach therefore disappear on the taxonomic family level.

However, both database approaches showed little overlap with the camera trap detections, even when it comes to only non-fish vertebrate taxa. This can partially be explained by the absence of detection of amphibian and reptile species by the camera trap. While eDNA methods should always be seen as a complementary approach to camera trapping or other traditional biodiversity monitoring approaches rather than a substitute, sampling different environmental materials such as soil, sediment, air, or blood-feeding insects could further increase the number of detected taxa. We, however, argue that the incompleteness of genetic reference databases might remain the largest limitation of eDNA-based approaches—especially when applied to highly biodiverse and/or unexplored regions—, and that serious *in situ* efforts to complete genetic databases **[26]** can help close the gap between traditional and genetics-based biodiversity monitoring approaches.

In field conditions, where access to high-performance computing may be limited, smaller, customized databases can be preferable to full public databases such as NCBI. This possible reduction of database sizes for plausible species detection importantly holds promise for data analysis in the field by substantially decreasing the computational memory requirements of taxonomic assignments; this aspect is especially relevant in combination with portable genomic technology where not only nanopore sequencing but also subsequent data interpretation should be feasible in remote areas and LMICs. However, our results highlight the risks associated with using reduced and/or incomplete databases. A reduced database may lead to misassignment of OTUs to closely related species, resulting in false detections [39]. This issue is particularly pronounced when using short genetic markers and broad taxonomic primers, which may lack the resolution needed to distinguish species accurately. Implementing strict sequence identity thresholds (e.g., 99%) between query and reference sequences can mitigate this problem, as can using longer genetic markers, which improve taxonomic resolution and reduce false positives [21].

Sample contamination can easily cause uncertainty in any eDNA study results. Our results showed several species being detected in a high number of samples including negative controls. For example, hippopotamus DNA was present in several samples and negative controls; while it was also captured by camera trapping, its occurrence in the negative controls cannot exclude the possibility of DNA contamination across samples. Similarly, the rainbow trout *Oncorhynchus mykiss* was detected in several samples and negative controls; while it does not naturally occur in the studied area, it has been reported as an invasive species in Zambia—and ruling out cross-contamination in such as case would be of utmost importance. Such DNA contamination remains a difficult challenge for any eDNA study, but especially in field laboratory conditions where access to clean space and sufficient equipment and reagents for spatial protocol separation is reduced. Additionally, *in situ* laboratory conditions can often imply the presence of wild species that can easily become sources of false-positive detections.

To finally make our eDNA approach as portable and cost-effective as possible, we further showed that simple store-bought coffee filters at USD 0.035 per piece led to similar biodiversity assessments as state-of-the-art eDNA filters at USD 10 per piece. We, however, also realized that the coffee filters were difficult to handle and easy to contaminate, so they might not constitute the perfect solution for non-expert users. While impossible to fully eliminate, especially in provisional laboratory conditions, the risk of contamination should be prioritized when applying eDNA approaches. We therefore argue that, if at all possible, eDNA collection and processing methods that reduce sample manipulation should be preferred despite potentially higher cost [46].

Supplementary Material

All supplementary material is currently available here. The code used for this study is available at: https://github.com/dmgr90/eDNA-Zambia-1

Data Accessibility Statement

All sequencing data is deposited in the NCBI Sequence Read Archive (SRA; accession number: PRJNA1195924).

Acknowledgements and Benefit-Sharing Statement

We thank the Department of National Parks and Wildlife for this fruitful collaboration. This research was carried out within and out of Zambia with their authorization under the 2023 research permit (Reference number: NPW/8/27/1).

References

1. Butchart, S. H. M., Walpole, M., Collen, B., van Strien, A., Scharlemann, J. P. W., Almond, R. E. A., Baillie, J. E. M., Bomhard, B., Brown, C., Bruno, J., Carpenter, K. E., Carr, G. M., Chanson, J., Chenery, A. M., Csirke, J., Davidson, N. C., Dentener, F., Foster, M., Galli, A., … Watson, R. 2010;. Global Biodiversity: Indicators of Recent Declines. Science, 328(5982), 1164–1168. doi: 10.1126/science.118751

2. Kindsvater, H. K., Dulvy, N. K., Horswill, C., Juan-Jordá, M.-J., Mangel, M., & Matthiopoulos, J. 2018;. Overcoming the Data Crisis in Biodiversity Conservation. Trends in Ecology & Evolution, 33(9), 676–688. doi: 10.1016/j.tree.2018.06.00

3. Vié, J.-C., Hilton-Taylor, C., & Stuart, S. N. 2009;. Wildlife in a changing world: An analysis of the 2008 IUCN red list of threatened species. IUCN ; Lynx Edicions

4. Lewis, D. 2019;. Rare bird's detection highlights promise of 'environmental DNA'. Nature, 575(7783), 423–424. doi: 10.1038/d41586-019-03522-

5. Matthias, L., Allison, M. J., Maslovat, C. Y., Hobbs, J., & Helbing, C. C. 2021;. Improving ecological surveys for the detection of cryptic, fossorial snakes using eDNA on and under artificial cover objects. Ecological Indicators, 131, 108187. doi: 10.1016/j.ecolind.2021.10818

6. Mizumoto, H., Mitsuzuka, T., & Araki, H. 2020;. An Environmental DNA Survey on Distribution of an Endangered Salmonid Species, Parahucho perryi, in Hokkaido, Japan. Frontiers in Ecology and Evolution, 8, 569425. doi: 10.3389/fevo.2020.56942

7. Itakura, H., Wakiya, R., Yamamoto, S., Kaifu, K., Sato, T., & Minamoto, T. 2019;. Environmental DNA analysis reveals the spatial distribution, abundance, and biomass of

Japanese eels at the river-basin scale. Aquatic Conservation: Marine and Freshwater Ecosystems, 29(3), 361–373. doi: 10.1002/aqc.3058

8. Nardi, C. F., Fernández, D. A., Vanella, F. A., & Chalde, T. 2019;. The expansion of exotic Chinook salmon (Oncorhynchus tshawytscha) in the extreme south of Patagonia: An environmental DNA approach. Biological Invasions, 21(4), 1415–1425. doi: 10.1007/s10530-018-1908-8

9. Sales, N. G., Wangensteen, O. S., Carvalho, D. C., Deiner, K., Præbel, K., Coscia, I., McDevitt, A. D., & Mariani, S. 2021;. Space-time dynamics in monitoring neotropical fish communities using eDNA metabarcoding. Science of The Total Environment, 754, 142096. doi: 10.1016/j.scitotenv.2020.142096

10. Fisher, B., & Christopher, T. 2007;. Poverty and biodiversity: Measuring the overlap of human poverty and the biodiversity hotspots. Ecological Economics, 62(1), 93–101. doi: 10.1016/j.ecolecon.2006.05.020

11. Urban, L., Perlas, A., Francino, O., Martí-Carreras, J., Muga, B. A., Mwangi, J. W., Boykin Okalebo, L., Stanton, J. L., Black, A., Waipara, N., Fontsere, C., Eccles, D., Urel, H., Reska, T., Morales, H. E., Palmada-Flores, M., Marques-Bonet, T., Watsa, M., Libke, Z., ... Van Oosterhout, C. 2023;. Real-time genomics for One Health. Molecular Systems Biology, e11686. doi: 10.15252/msb.20231168

12. Watsa, M., Erkenswick, G. A., Pomerantz, A., & Prost, S. 2020;. Portable sequencing as a teaching tool in conservation and biodiversity research. PLOS Biology, 18(4), e3000667. doi: 10.1371/journal.pbio.3000667

13. Convention on International Trade in Endangered Species of Wild Fauna and Flora | CITES. (n.d.). Retrieved December 6, 2024, from https://cites.org/eng/disc/text.php

14. The Nagoya Protocol on Access and Benefit-sharing. (n.d.). Retrieved December 6, 2024, from https://www.cbd.int/ab

15. CARE Principles—Global Indigenous Data Alliance. (n.d.). Retrieved December 6, 2024, from https://www.gida-global.org/care

16. Urban, L., Miller, A. K., Eason, D., Vercoe, D., Shaffer, M., Wilkinson, S. P., Jeunen, G.-J., Gemmell, N. J., & Digby, A. 2022;. Non-invasive real-time genomic monitoring of the critically endangered kākāpō [Preprint]. Genomics. doi: 10.1101/2022.11.14.516431

17. Baloğlu, B., Chen, Z., Elbrecht, V., Braukmann, T., MacDonald, S., & Steinke, D. 2021;. A workflow for accurate metabarcoding using nanopore MinION sequencing. Methods in Ecology and Evolution, 12(5), 794–804. doi: 10.1111/2041-210X.13561

18. Bludau, D., Sieber, G., Shah, M., Deep, A., Boenigk, J., & Beisser, D. 2025;. Breaking the Standard: Can Oxford Nanopore Technologies Sequencing Compete With Illumina in Protistan Amplicon Studies? Environmental DNA, 7(2), e70084. doi: 10.1002/edn3.70084

19. Van Der Reis, A. L., Beckley, L. E., Olivar, M. P., & Jeffs, A. G. 2023;. Nanopore short-read sequencing: A quick, cost-effective and accurate method for DNA metabarcoding. Environmental DNA, 5(2), 282–296. doi: 10.1002/edn3.374

20. Egeter, B., Veríssimo, J., Lopes-Lima, M., Chaves, C., Pinto, J., Riccardi, N., Beja, P., & Fonseca, N. A. 2021;. Metabarcoding with MinION: Speeding up the detection of invasive

aquatic species using environmental DNA and nanopore sequencing. ARPHA Conference Abstracts, 4, e65036. doi: 10.3897/aca.4.e6503

21. Doorenspleet, K., Jansen, L., Oosterbroek, S., Kamermans, P., Bos, O., Wurz, E., Murk, A., & Nijland, R. 2025;. The Long and the Short of It: Nanopore-Based eDNA Metabarcoding of Marine Vertebrates Works; Sensitivity and Species-Level Assignment Depend on Amplicon Lengths. Molecular Ecology Resources, 25(4), e14079. doi: 10.1111/1755-0998.1407

22. Government of the Republic of Zambia. 2015;. Zambia's second national biodiversity strategy and action plan (NBSAP-2) 2015–2025. Ministry of Lands, Natural Resources and Environmental Protection.

23. Critical Ecosystem Partnership Fund (CEPF). Final Project Completion Report: Project Number SG68954. 2 017. Available from: https://www.cepf.net/sites/default/files/final-report-sg68954.pdf

24. Center for Large Landscape Conservation (CLLC). 2020;. Zambia FOCUS-BRI Report. Retrieved from https://largelandscapes.org/wp-content/uploads/Zambia-FOCUS-BRI.pdf

25. Riaz, T., Shehzad, W., Viari, A., Pompanon, F., Taberlet, P., & Coissac, E. 2011;. ecoPrimers: Inference of new DNA barcode markers from whole genome sequence analysis. Nucleic Acids Research, 39(21), e145–e145. doi: 10.1093/nar/gkr732

26. Srivathsan, A., Feng, V., Suárez, D., Emerson, B., & Meier, R. 2023;. Rapid species discovery and identification with real-time barcoding facilitated by ONTbarcoder 2.0 and Oxford Nanopore R10.4 [Preprint]. Ecology. doi: 10.1101/2023.06.26.546538

27. Martin, M. 2011; Cutadapt Removes Adapter Sequences from High-Throughput Sequencing Reads. EMBnet Journal, 17, 10-12

28. Rognes, T., Flouri, T., Nichols, B., Quince, C., & Mahé, F. 2016;. VSEARCH: A versatile open source tool for metagenomics. PeerJ, 4, e2584. doi: 10.7717/peerj.258

29. Bushnell, B. 2014;. BBTools software package. https://jgi.doe.gov/data-and-tools/bbtools/

30. Camacho, C., Coulouris, G., Avagyan, V., Ma, N., Papadopoulos, J., Bealer, K., & Madden, T. L. 2009;. BLAST+: Architecture and applications. BMC Bioinformatics, 10:421. doi: 10.1186/1471-2105-10-42

31. Urban, L., Holzer, A., Baronas, J. J., Hall, M. B., Braeuninger-Weimer, P., Scherm, M. J., Kunz, D. J., Perera, S. N., Martin-Herranz, D. E., Tipper, E. T., Salter, S. J., & Stammnitz, M. R. 2021;. Freshwater monitoring by nanopore sequencing. eLife, 10, e61504. doi: 10.7554/eLife.61504

32. Bista, I., Carvalho, G. R., Tang, M., Walsh, K., Zhou, X., Hajibabaei, M., Shokralla, S., Seymour, M., Bradley, D., Liu, S., Christmas, M., & Creer, S. 2018;. Performance of amplicon and shotgun sequencing for accurate biomass estimation in invertebrate community samples. Molecular Ecology Resources, 18(5), 1020–1034. doi: 10.1111/1755-0998.1288

33. Deagle, B. E., Jarman, S. N., Coissac, E., Pompanon, F., & Taberlet, P. 2014;. DNA metabarcoding and the cytochrome c oxidase subunit I marker: Not a perfect match. Biology Letters, 10(9), 20140562. doi: 10.1098/rsbl.2014.056

34. Jo, T., Murakami, H., Masuda, R., Sakata, M. K., Yamamoto, S., & Minamoto, T. 2017;. Rapid degradation of longer DNA fragments enables the improved estimation of distribution

and biomass using environmental DNA. Molecular Ecology Resources, 17(6). doi: 10.1111/1755-0998.12685

35. Deiner, K., Bik, H. M., Mächler, E., Seymour, M., Lacoursière-Roussel, A., Altermatt, F., Creer, S., Bista, I., Lodge, D. M., Vere, N., Pfrender, M. E., & Bernatchez, L. 2017;. Environmental DNA metabarcoding: Transforming how we survey animal and plant communities. Molecular Ecology, 26(21), 5872–5895. doi: 10.1111/mec.14350

36. Li, F., Zhang, Y., Altermatt, F., Zhang, X., Cai, Y., & Yang, Z. 2022;. Gap analysis for DNA-based biomonitoring of aquatic ecosystems in China. Ecological Indicators, 137, 108732. doi: 10.1016/j.ecolind.2022.10873

37. Marques, V., Milhau, T., Albouy, C., Dejean, T., Manel, S., Mouillot, D., & Juhel, J. 2021;. GAPeDNA: Assessing and mapping global species gaps in genetic databases for eDNA metabarcoding. Diversity and Distributions, 27(10), 1880–1892. doi: 10.1111/ddi.13142

38. Supple, M. A., & Shapiro, B. 2018;. Conservation of biodiversity in the genomics era. Genome Biology, 19(1), 131. doi: 10.1186/s13059-018-1520-3

39. Keck, F., Couton, M., & Altermatt, F. 2023;. Navigating the seven challenges of taxonomic reference databases in metabarcoding analyses. Molecular Ecology Resources, 23(4), 742–755. doi: 10.1111/1755-0998.13746

40. Mugnai, F., Costantini, F., Chenuil, A., Leduc, M., Gutiérrez Ortega, J. M., & Meglécz, E. 2023;. Be positive: Customized reference databases and new, local barcodes balance false taxonomic assignments in metabarcoding studies. PeerJ, 11, e14616. doi: 10.7717/peerj.1461

41. Allen, M. C., Kwait, R., Vastano, A., Kisurin, A., Zoccolo, I., Jaffe, B. D., Angle, J. C., Maslo, B., & Lockwood, J. L. 2023;. Sampling environmental DNA from trees and soil to detect cryptic arboreal mammals. Scientific Reports, 13(1), 180. doi: 10.1038/s41598-023-27512-8

42. Lozano Mojica, J. D., & Caballero, S. 2021;. Applications of eDNA Metabarcoding for Vertebrate Diversity Studies in Northern Colombian Water Bodies. Frontiers in Ecology and Evolution, 8, 617948. doi: 10.3389/fevo.2020.61794

43. Lynggaard, C., Bertelsen, M. F., Jensen, C. V., Johnson, M. S., Frøslev, T. G., Olsen, M. T., & Bohmann, K. 2022;. Airborne environmental DNA for terrestrial vertebrate community monitoring. Current Biology, 32(3), 701-707.e5. doi: 10.1016/j.cub.2021.12.01

44. Polling, M., Buij, R., Laros, I., & De Groot, G. A. 2024;. Continuous daily sampling of airborne eDNA detects all vertebrate species identified by camera traps. Environmental DNA, 6(4), e591. doi: 10.1002/edn3.59

45. Meglécz, E. 2023;. COInr and mkCOInr: Building and customizing a nonredundant barcoding reference database from BOLD and NCBI using a semi-automated pipeline. Molecular Ecology Resources, 23(4), 933–945. doi: 10.1111/1755-0998.1375

46. Macher, T.-H., Arle, J., Beermann, A. J., Frank, L., Hupało, K., Koschorreck, J., Schütz, R., & Leese, F. 2024;. Is it worth the extra mile? Comparing environmental DNA and RNA metabarcoding for vertebrate and invertebrate biodiversity surveys in a lowland stream. PeerJ, 12, e18016. doi: 10.7717/peerj.1801

Figure 1

Click here to access/download;Figure;Figure 1.tif

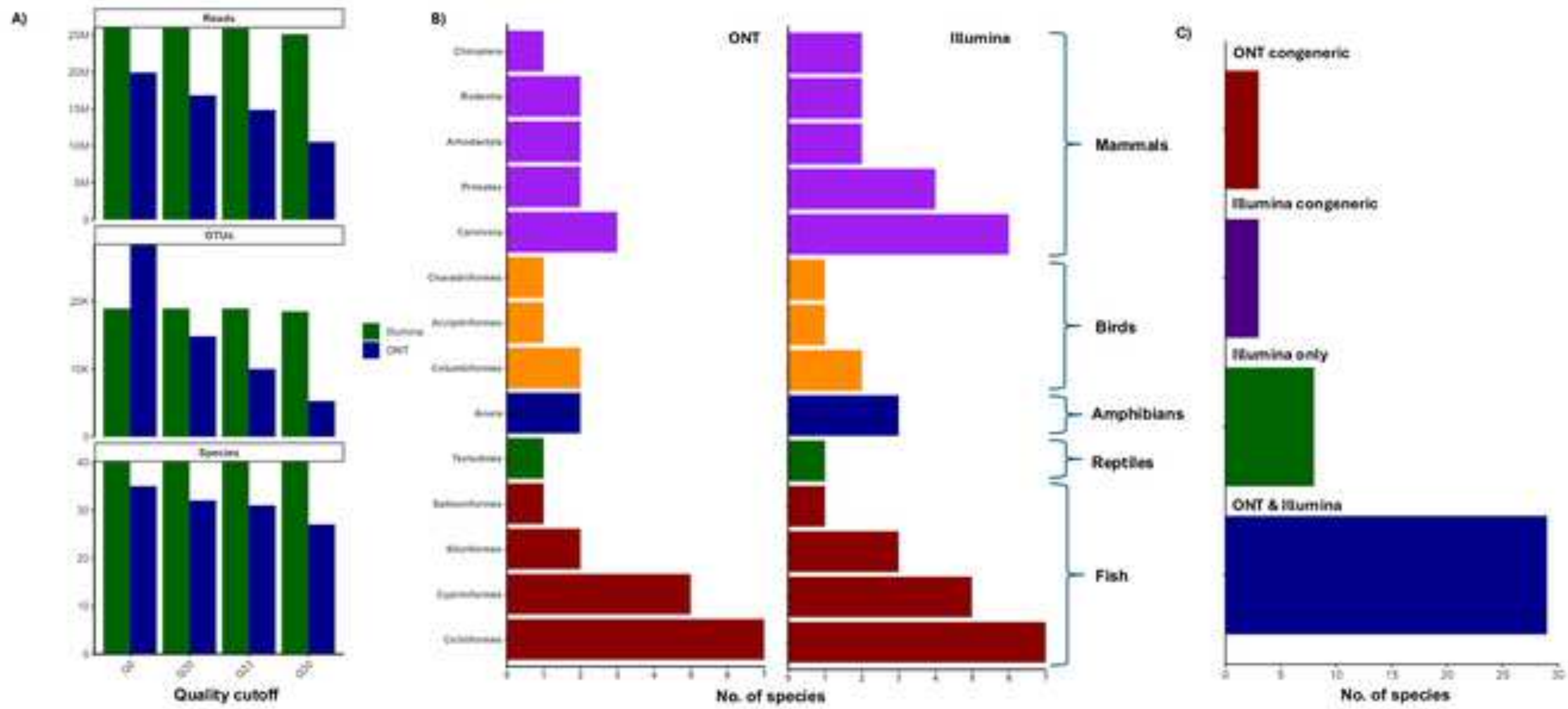

Figure 2

Click here to access/download;Figure;Figure 2.tiff

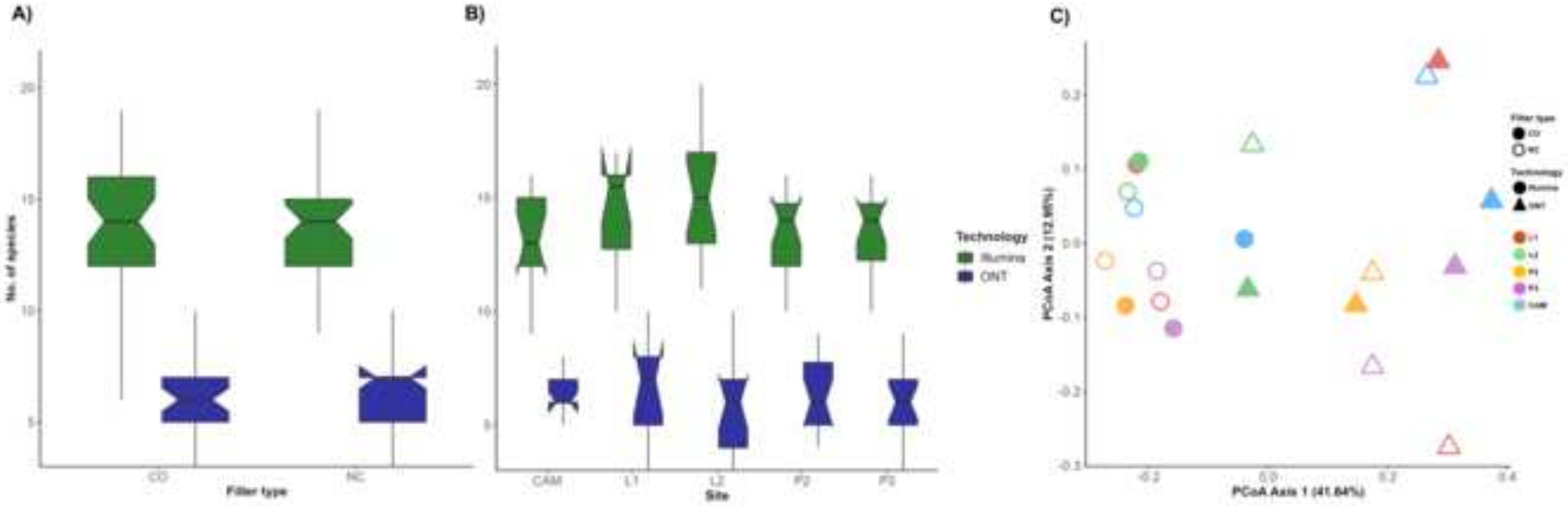

Figure 3

Click here to access/download;Figure;Figure 3.tiff

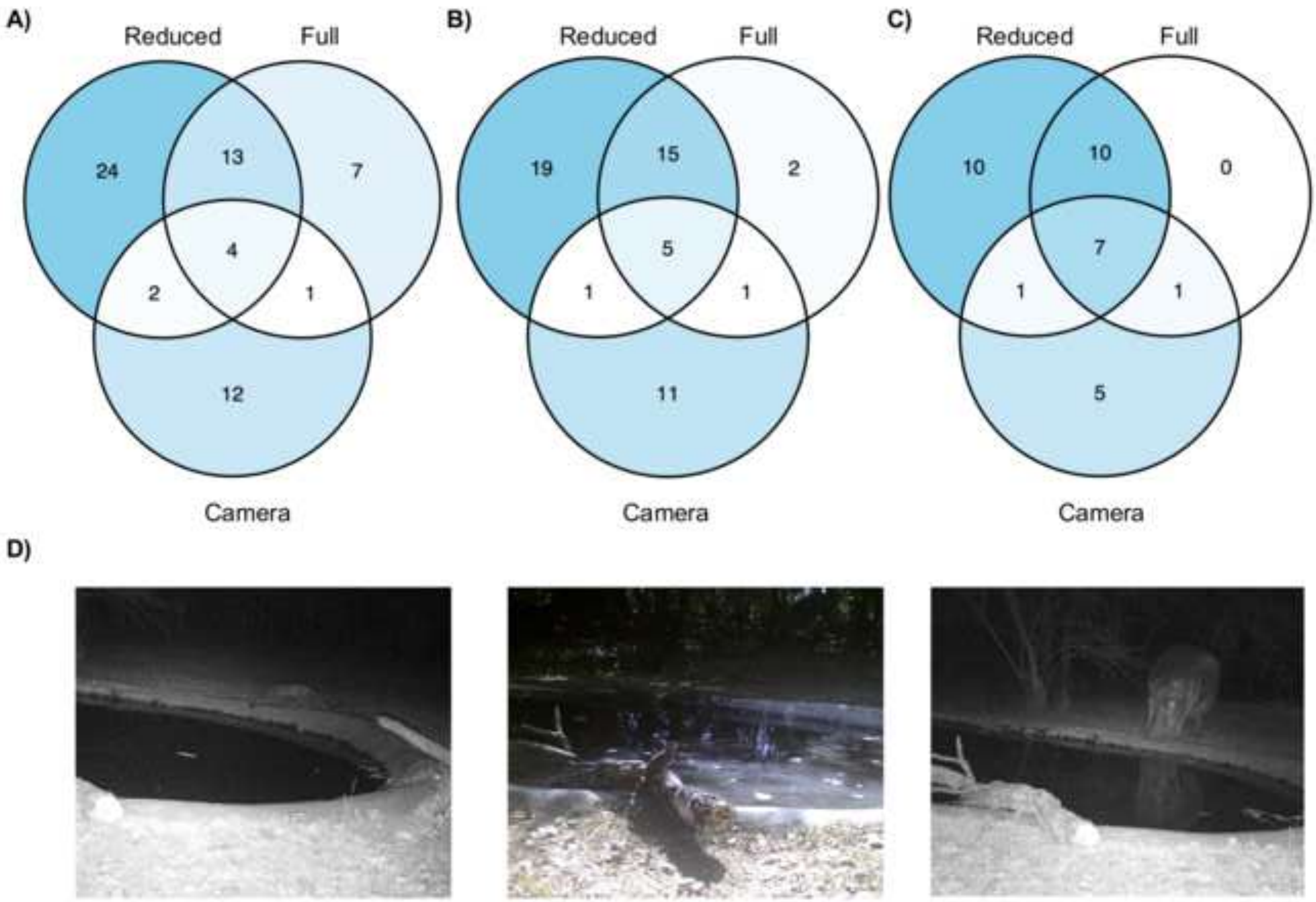

Table 1 Click here to access/download;Figure;Table 1.tiff

Species	Family	Order	PCR's (ONT)	PCR's (Illumina)	Technology	ONT neg.	Illimina neg.
Acinonyx jubatus	Felidae	Carnivora	0	2	Illumina		
Afronycteris nana	Vespertilionidae	Chiroptera	1	7	ONT , Illumina		
Aquila nipalensis	Accipitridae	Accipitriformes	3	68	ONT , Illumina		Yes
Astatotilapia calliptera	Cichlidae	Perciformes	23	23	ONT , Illumina	Yes	Yes
Cercopithecus ascanius	Cercopithecidae	Primates	0	1	Illumina		
Cercopithecus mitis	Cercopithecidae	Primates	2	6	ONT , Illumina		Yes
Charadrius mongolus	Charadriidae	Charadriiformes	1	7	ONT , Illumina		
Chlorocebus aethiops	Cercopithecidae	Primates	5	39	ONT , Illumina	Yes	Yes
Chrysichthys brachynema	Claroteidae	Siluriformes	4	74	ONT , Illumina		Yes
Clarias gariepinus	Clariidae	Siluriformes	75	82	ONT , Illumina	Yes	Yes
Columba livia	Columbidae	Columbiformes	1	11	ONT , Illumina		
Coptodon rendalli	Cichlidae	Perciformes	4	4	ONT , Illumina		Yes
Cyprichromis leptosoma	Cichlidae	Perciformes	0	1	Illumina		Yes
Cyprinus carpio	Cyprinidae	Cypriniformes	40	60	ONT , Illumina	Yes	Yes
Enteromius eutaenia	Cyprinidae	Cypriniformes	1	41	ONT , Illumina		Yes
Enteromius trimaculatus	Cyprinidae	Cypriniformes	1	14	ONT , Illumina		
Epomophorus minor	Pteropodidae	Chiroptera	0	1	Illumina		
Galago moholi	Galagidae	Primates	0	2	Illumina		
Galerella sanguinea	Herpestidae	Carnivora	1	3	ONT , Illumina		
Genetta genetta	Viverridae	Carnivora	2	14	ONT , Illumina		
Graphiurus kelleni	Gliridae	Rodentia	0	4	Illumina		
Graphiurus lorraineus	Gliridae	Rodentia	1	1	ONT , Illumina		
Graphiurus microtis	Gliridae	Rodentia	1	0	ONT		
Helogale parvula	Herpestidae	Carnivora	6	78	ONT , Illumina		Yes
Herpestes ichneumon	Herpestidae	Carnivora	0	2	Illumina		
Hippopotamus amphibius	Hippopotamidae	Artiodactyla	35	81	ONT , Illumina	Yes	Yes
Hyperolius viridiflavus	Hyperoliidae	Anura	0	2	Illumina		
Labeo congoro	Cyprinidae	Cypriniformes	4	30	ONT , Illumina		Yes
Labeo cylindricus	Cyprinidae	Cypriniformes	6	24	ONT , Illumina		Yes
Oncorhynchus mykiss	Salmonidae	Salmoniformes	7	57	ONT , Illumina	Yes	Yes
Oreochromis aureus	Cichlidae	Perciformes	2	3	ONT , Illumina		
Oreochromis macrochir	Cichlidae	Perciformes	79	82	ONT , Illumina	Yes	Yes
Oreochromis niloticus	Cichlidae	Perciformes	58	54	ONT , Illumina	Yes	Yes
Oreochromis tanganicae	Cichlidae	Perciformes	37	70	ONT , Illumina	Yes	Yes
Pelusios sinuatus	Pelomedusidae	Testudines	1	1	ONT , Illumina		
Phrynobatrachus natalensis	Phrynobatrachidae	Anura	0	2	Illumina		
Rhynchogale melleri	Herpestidae	Carnivora	0	3	Illumina		
Streptopelia capicola	Columbidae	Columbiformes	1	1	ONT , Illumina		
Synodontis polli	Mochokidae	Siluriformes	0	1	Illumina		
Tragelaphus scriptus	Bovidae	Artiodactyla	1	0	ONT		
Tragelaphus strepsiceros	Bovidae	Artiodactyla	0	3	Illumina		
Tropheus moorii	Cichlidae	Perciformes	75	82	ONT , Illumina	Yes	Yes
Xenopus laevis	Pipidae	Anura	3	0	ONT		
Xenopus muelleri	Pipidae	Anura	23	82	ONT , Illumina	Yes	Yes

Supplementary Table 1

Click here to access/download
Supporting Information
supp-1.txt

Supplementary Table 2

Click here to access/download
Supporting Information
supp-2.txt

Supplementary Table 3

Click here to access/download
Supporting Information
supp-3.txt

Supplementary Table 4

Click here to access/download
Supporting Information
supp-4.csv

Supplementary Table 5

Click here to access/download
Supporting Information
supp-5.csv

Supplementary Table 6

Click here to access/download
Supporting Information
supp-6.txt

Preprint

Click here to access/download
Preprint PDF
pone-649756.pdf

Click here to access/download
Preprint PDF
msfile-1.pdf
