# Supplementary material for: Evaluation of nanopore sequencing for increasing accessibility of eDNA studies in biodiverse countries
Source: PLoS One. 2025 Oct 16;20(10):e0333994. doi: 10.1371/journal.pone.0333994 (PMC12530551; doi:10.1371/journal.pone.0333994)
Supplement: Attachment — Submitted filename: Response to Reviewers.pdf [file pone.0333994.s006.pdf]

Munich, September 4th, 2025

Mizanur Rahman, Ph.D.
Academic Editor
PLOS ONE
Thriges Pl. 8, 5000 Odense C, Denmark

Dear Editor and Reviewers,

We would like to thank you for the constructive and insightful comments on our manuscript "*Evaluation of nanopore sequencing for increasing accessibility of eDNA studies in biodiverse countries*". The comments have been very helpful in improving the quality and clarity of the manuscript. While we provide a detailed point-by-point response below, we here summarize the additional major changes of the manuscript (which are also highlighted by yellow background in the version of the manuscript with tracked changes):

1) We have integrated completely new results from the *in-situ* application of our nanopore sequencing-based mobile eDNA laboratory in Zambia, showcasing that eDNA approaches are feasible to conduct in the field from sample collection to data analysis.
2) We have added eDNA assessments by an additional primer set throughout the manuscript, targeting the mammalian 16S rRNA gene in addition to the vertebrate 12S rRNA gene, to increase the number of potential species detections. To give more context of the study area and species ecology, we included a map of the study area to provide clearer geographic context and also discuss the ecology of the detected species in more detail. We, however, emphasize that the focus of our manuscript is on the comparison of established and portable sequencing approaches for global eDNA research.
3) We removed the following results from the manuscript to increase clarity:
    a. We removed all fish species detections and now focus on primary terrestrial vertebrate taxa since the accuracy of fish identifications is difficult to validate due to a lack of knowledge about the regional fish diversity.
    b. We additionally removed all results obtained through coffee filters since they introduced substantial contamination; for the *in-situ* application, we instead introduced a novel sampling technique that minimizes the risk of contamination. To be stringent throughout the manuscript, we removed any species detections that were also found in the respective negative controls.
    c. We removed the comparison between the full NCBI nucleotide and custom databases and instead used the curated MIDORI2 database which

combines taxonomic coverage with a compact size so that it can be run on a portable laptop.

Comments from the Academic Editor:

If applicable, we recommend that you deposit your laboratory protocols in protocols.io to enhance the reproducibility of your results. Protocols.io assigns your protocol its own identifier (DOI) so that it can be cited independently in the future. For instructions see: https://journals.plos.org/plosone/s/submission-guidelines#loc-laboratory-protocols. Additionally, PLOS ONE offers an option for publishing peer-reviewed Lab Protocol articles, which describe protocols hosted on protocols.io. Read more information on sharing protocols at https://plos.org/protocols?utm_medium=editorial-email&utm_source=authorletters&utm_campaign=protocols.

Response:

We ensured that all laboratory protocols are described in sufficient detail in our manuscript, and as we will publish the manuscript in open-access format, we believe that this is sufficient at this point.

Comments from Reviewer #1:

Current manuscript is well defined and satisfactory, however I would suggest some minor changes as required to be considered below,
Abstract satisfactory
Introduction satisfactory
Methodology satisfactory, but must add the citation for defining the methodology adopted by you for this article
Results and discussion well defined and satisfactory, but add the forward and reverse sequence of each species identified by you and compare them with 5 previously identified species from NCBI data base as you mentioned in your methodology for comparison between your eDNA technique with them. Define the percentage of matching with previous barcoding of each species with your data.
If possibility compare your major outcomes of usage of eDNA technology for vertebrates species with other DNA barcoding technologies.
Also add the morphological identification characters of your each identify species and compare it with previous publication, so middle income countries researchers also used them for their regional identification of fauna found in their freshwater resources

Wish you best of luck

<u>Response:</u>

We thank the Reviewer for these positive comments. We added references to all relevant previous work. As detailed above, we now use the curated MIDORI2 database instead of the full NCBI, which enabled taxonomic assignment analyses on a portable laptop on site in Zambia.

To clarify our work to the reviewer, we did not perform any barcoding work, but metabarcoding, which relies on taxonomic classification of OTUs after clustering of sequences. We can therefore not provide the forward and reverse sequence on a per-species level. Using the MIDORI2 database, which is widely used in metabarcoding applications for eDNA, we then accepted species matches at a stringent high-identity threshold (98%, with at least 80% query coverage) and additionally limited the reported species detections to local Zambian species. We describe all of this in details in the manuscript's methods.

As we have not performed barcoding of animals, but metabarcoding of non-invasive samples, morphological identification is unfortunately not feasible.

Comments from Reviewer #2:

The study by Dr Gygax et al., is interesting in that it focuses on a modern theme whose application and accuracy still require years of work before being fully effective. Therefore, any exploratory contribution in this area is essential.

The study focused on comparing the latest portable nanopore sequencing methods with established Illumina sequencing for vertebrate eDNA metabarcoding of Zambian water samples; moreover, a cost-effective evaluation of different methods of water filtration approaches, and database settings was provided.

The manuscript is well written and organized, although a minimum of revision of the English by a native speaker could improve its overall fluency. The use of figures and tables is appropriate.

The introduction lacks an important premise regarding the effectiveness of the eDNA method in general. The limits connected to the taxonomic assignment of the references

present today in the databases are not mentioned, among other things, of little quantity, as evidenced by the authors speaking individually of the studied taxonomic groups. In addition to a quantitative argument, an not non-negligible problem also concerns the fact that the annotations in most public databases are made without any taxonomic revision, and the classical morphological identification of the species can sometimes be doubtful, especially when talking about cryptic or elusive species. The use of eDNA at present does not solve this problem; on the contrary, it probably aggravates it, assigning OTUs as sequences that may not be exactly the species noted. In this light, could the comparison of different sequencing methods and instrumentation have potential implications? Or does it remain a basic limitation of the eDNA technique, for the moment, not unlimited? This discourse should be introduced and argued, with the support of appropriate consequences.

Response:

We appreciate the reviewer's concern; we made sure to include an overview of the effectiveness of past eDNA applications in the Introduction. We fully agree that database incompleteness remains a major challenge for eDNA and metabarcoding approaches. We emphasize this point in the manuscript and highlight the need to generate additional reference sequences (285). Our results illustrate this clearly using Zambia as an example, where a large proportion of vertebrate groups currently lack genetic references: Our manuscript firstly assesses the genetic sequence representation of Zambian primary terrestrial vertebrates (lines 275-282, 383-384). We then discuss the effect of database incompleteness on metabarcoding studies (lines 385–393), providing context for the strengths and limitations of our approach. We here clarify that database incompleteness poses a general problem for eDNA research, independently of the sequencing method.

The contamination of negative samples is reported in the results and briefly discussed. At present, it is not very clear what this contamination may be due to, and the authors do not explain well how the repetitive presence of such contamination in a significant number of samples influenced the study, casting doubt on the potential bias of the same.

It is not clear from the tables in the results sections if this contamination was equal for both extraction methods; this could be an interesting data to show and argue better. The effect that this contamination may have had on the study, and the authors' idea regarding its origin, should be explained and better argued.

Response:

We first removed all results obtained through coffee filters from our study since they introduced substantial cross-sample contamination; for the *in situ* application

of our approach, we instead introduce a novel sampling technique that minimizes the risk of contamination. To be stringent throughout the manuscript, we removed any local species detections that were also found in the remaining negative controls; all details are provided in the manuscript (lines 215, 283-285, 319).

We additionally observed contamination by human DNA, which is a common issue with vertebrate primers due to their amplification of human sequences. We therefore applied human-blocking primers during the new *in situ* application of the study, which significantly reduced human DNA contamination. All protocols to reduce the risk of human DNA amplification are provided in the manuscript (lines 236-242, 252-263).

Another important limitation of the study concerns the comparative methods of analysis of aquatic diversity, mainly fish, which are excluded from most of the effects of the study, and trapping is used as a method to normalize data from eDNA monitoring. But what are the standard methods for monitoring these organisms in the studied area? This is not mentioned, as well as the decision not to apply these methods to make the effects of the study wider. From this point of view, it is a pity that we cannot be relevant to an abundance of vertebrate species.

Best regards

Response:

We agree with the limitations detailed by the reviewer. To ensure reliability of our results, we removed all fish species detections and now specify in the manuscript that we focus on primary terrestrial vertebrate taxa—since the accuracy of fish identifications is difficult to validate due to a lack of knowledge about the regional fish diversity. The focus of our manuscript was further the comparison of established and portable sequencing approaches for eDNA-based detection, and their comparison with conventional camera trapping—and therefore with a focus on primary terrestrial vertebrates which can also be captured by camera trapping.

Comments from Reviewer #3:

This manuscript presents a timely and relevant evaluation of nanopore sequencing as a tool for environmental DNA (eDNA)-based biodiversity monitoring in the Luangwa Valley, Zambia. The authors provide a comprehensive comparison of nanopore and Illumina platforms, exploring aspects such as cost-effective filtration methods, taxonomic assignment strategies, and field applicability. The methodological framework is robust and responds to current needs for accessible genomic tools in low- and middle-income

countries.
However, while technically rigorous, the manuscript does not sufficiently articulate the ecological significance of its findings. The framing remains primarily methodological, with limited integration of ecological context, species behavior, or environmental gradients. To increase the impact and clarity of the manuscript (especially for an audience concerned with freshwater biodiversity dynamics) major revisions are needed to anchor the research in ecological theory and regional ecosystem understanding. The discussion should go beyond sequence performance metrics to explore how findings inform our knowledge of biodiversity, species distribution, and ecosystem monitoring practices.

Response:

While we agree that a discussion of the ecological significance could enrich our study, our focus was primarily a technical comparison between established and portable sequencing technologies for eDNA-based biodiversity assessments in comparison to conventional camera trapping approaches. We hope that we could emphasize this focus of the manuscript by integrating completely new results from the actual *in situ* application of our nanopore sequencing-based mobile eDNA protocols in Zambia, showcasing that eDNA approaches are now feasible to conduct in the field from sample collection to data analysis. As we, however, had access to eDNA- and camera trapping-based detections of primary terrestrial vertebrate species, we at least discuss the potential ecological reasons for these discrepancies in more detail (lines 394–416).

Major comments

Abstract
Your abstract does not highlight any values. You need to highlight the values for better understanding.

Response:

We now highlight that the focus of our study was the *in situ* application of eDNA by portable genomic technology: "We finally demonstrate that our entire eDNA workflow can be successfully implemented in a mobile laboratory under remote field conditions by completing all steps—from sample collection to data analysis—within the Luambe National Park in Zambia. This approach has important implications for capacity building in LMICs and for overcoming limitations associated with sample export."

Introduction

The current introduction lacks a clearly articulated ecological rationale. The focus is on technical limitations in LMICs without linking these constraints to ecological or conservation challenges specific to African savannah ecosystems. Recommendation : Reformulate the objectives to explicitly include ecological hypotheses, e.g., “This study investigates whether nanopore sequencing can effectively detect vertebrate biodiversity in dynamic subtropical freshwater systems, with implications for ecological monitoring in seasonally variable habitats.” Introduce the concept of ecological detectability (species behavior, habitat use, waterbody permanence) and refer to studies that illustrate biodiversity dynamics in similar African wetland systems.

Response:

As detailed above, our manuscript is focused on comparisons between technologies, and not on any ecological hypotheses. We now further emphasize the goal of this study in the Introduction, and discuss finding of potential ecological significance in our Discussion only to the extent that it can potentially explain discrepancies in species detections between the different technologies. Our benchmarking study as well as our *in situ* application at this point lack the sample size to make statement on biodiversity dynamics; we have now ensured throughout the manuscript that we don’t make any such claims.

Materials and methods

Although the sampling locations are georeferenced and categorized (ponds vs river), there is no ecological description of these habitats. Please add a descriptive map of the study area.

Recommendation: Enrich the section with ecological characterizations of the sites (e.g., temporary vs permanent waterbodies, anthropogenic vs natural origin). These descriptions could frame subsequent interpretations of taxonomic detections, especially considering species habitat preferences and seasonal usage of aquatic environments by terrestrial fauna (e.g., hippos, amphibians).

Response:

We added a map marking the sampling site. We also included photographs of the sampling sites and specified if it was an artificial or natural water hole or riverside.

Discussion
The list and frequency of species detected are reported but not discussed in relation to their ecological traits or habitat use.
Recommendation : In this section, comment on the ecological roles and behaviors of key detected species (e.g., Hippopotamus amphibius as an aquatic-terrestrial connector, Xenopus species as indicators of water quality). Highlight how eDNA reveals cryptic taxa that may not be easily detected through camera trapping, especially amphibians and small mammals.

Response:

We included some of these discussion points (lines 402-416), thank you for your comments!

Minor comments
Lines 22–26, 29 and 76–80 : Remove the personal pronouns from the sentences.

Recommendation : For example, Line 22: This study compares the latest portable nanopore sequencing methods... Lines 70–75 : This is a very long sentence that is difficult to understand.

Response:

We note that the use of personal pronouns such as “we” is common in other PLoS ONE articles, and therefore consider it appropriate in the context of this manuscript. We shortened the long sentence, and made sure throughout the manuscript that sentences are concise.

Comments from Reviewer #4:

The study titled “Evaluation of nanopore sequencing for increasing accessibility of eDNA studies in biodiverse countries” was aimed at comparing the latest portable nanopore sequencing methods with established Illumina sequencing for vertebrate eDNA metabarcoding of Zambian water samples. A cost-effective versus established water filtration approaches was evaluated, and contrasted with a comprehensive, computationally intensive taxonomic database search with a streamlined manually curated database search. The study was well designed and thought out. I have appended below comments that may help improve the manuscript:

The introduction was well written, however there seem to be so many wordy sentences as most of the sentences were difficult to understands, I suggest breaking them up into sizeable texts that can easily be comprehended. Other few comments can be seen in the attached document.

Response:

We shortened sentences throughout the manuscript.

The materials and methods section were well written and structured, but I was expecting to a subsection dedicated to description of location and even the entire study area. I didn't see map of the sampled locations; this is key in such ecological studies.

Response:

We added a map marking the sampling site. We also included photographs of the sampling sites and specified if in was an artificial or natural water hole or riverside.

The results are okay; however, I found it difficult to see information on the figures clearly, this need to be recreated for visibility.

Response:

We changed all of the figures to increase clarity, and also to accommodate new data.

The discussion is okay, but there seem to be too much unnecessary information which made the discussion in some part redundant, so I would suggest you revisit this section and objectively discuss your key findings and reduce the excessive explanation of none relevant terms or concepts with regards to the topic of discussion.

Response:

We substantially revised the discussion to reduce redundancy and improve clarity. In addition, we have included new discussion sections addressing the newly added data.

We would again like to thank the editor and reviewers for the very helpful comments, and look forward to receiving further feedback.

With best regards,

Daniel Gygax & Lara Urban, on behalf of all authors
