# Supplementary material for: Evaluation of nanopore sequencing for increasing accessibility of eDNA studies in biodiverse countries
Source: PLoS One. 2025 Oct 16;20(10):e0333994. doi: 10.1371/journal.pone.0333994 (PMC12530551; doi:10.1371/journal.pone.0333994)
Supplement: Attachment — Submitted filename: Review of manuscript PONE-D-25-24113.docx [file pone.0333994.s004.docx]

**Manuscript title : "Evaluation of nanopore sequencing for increasing accessibility of eDNA studies in biodiverse countries"**

This manuscript presents a timely and relevant evaluation of nanopore sequencing as a tool for environmental DNA (eDNA)-based biodiversity monitoring in the Luangwa Valley, Zambia. The authors provide a comprehensive comparison of nanopore and Illumina platforms, exploring aspects such as cost-effective filtration methods, taxonomic assignment strategies, and field applicability. The methodological framework is robust and responds to current needs for accessible genomic tools in low- and middle-income countries.

However, while technically rigorous, the manuscript does not sufficiently articulate the ecological significance of its findings. The framing remains primarily methodological, with limited integration of ecological context, species behavior, or environmental gradients. To increase the impact and clarity of the manuscript (especially for an audience concerned with freshwater biodiversity dynamics) major revisions are needed to anchor the research in ecological theory and regional ecosystem understanding. The discussion should go beyond sequence performance metrics to explore how findings inform our knowledge of biodiversity, species distribution, and ecosystem monitoring practices.

**Major comments**

**Abstract**

Your abstract does not highlight any values. You need to highlight the values for better understanding.

**Introduction**

The current introduction lacks a clearly articulated ecological rationale. The focus is on technical limitations in LMICs without linking these constraints to ecological or conservation challenges specific to African savannah ecosystems.

Recommendation : Reformulate the objectives to explicitly include ecological hypotheses, e.g., “This study investigates whether nanopore sequencing can effectively detect vertebrate biodiversity in dynamic subtropical freshwater systems, with implications for ecological monitoring in seasonally variable habitats.” Introduce the concept of ecological detectability (species behavior, habitat use, waterbody permanence) and refer to studies that illustrate biodiversity dynamics in similar African wetland systems.

**Materials and methods**

Although the sampling locations are georeferenced and categorized (ponds vs river), there is no ecological description of these habitats. Please add a descriptive map of the study area.

Recommendation: Enrich the section with ecological characterizations of the sites (e.g., temporary vs permanent waterbodies, anthropogenic vs natural origin). These descriptions could frame subsequent interpretations of taxonomic detections, especially considering species habitat preferences and seasonal usage of aquatic environments by terrestrial fauna (e.g., hippos, amphibians).

**Discussion**

The list and frequency of species detected are reported but not discussed in relation to their ecological traits or habitat use.

Recommendation : In this section, comment on the ecological roles and behaviors of key detected species (e.g., *Hippopotamus amphibius* as an aquatic-terrestrial connector, *Xenopus* species as indicators of water quality). Highlight how eDNA reveals cryptic taxa that may not be easily detected through camera trapping, especially amphibians and small mammals.

**Minor comments**

Lines 22–26, 29 and 76–80 : Remove the personal pronouns from the sentences.

Recommendation : For example, Line 22: This study compares the latest portable nanopore sequencing methods...

Lines 70–75 : This is a very long sentence that is difficult to understand.

Recommendation : Split the text into two sentences.

**Final recommendation**

Minor revisions are required. Once these comments have been addressed, the manuscript may be published in PLOS ONE.
